# Chitosan-Based Nanoparticles for Intracellular Delivery of ISAV Fusion Protein cDNA into Melanoma Cells: A Path to Develop Oncolytic Anticancer Therapies

**DOI:** 10.1155/2020/8680692

**Published:** 2020-04-29

**Authors:** Claudia Robles-Planells, Giselle Sánchez-Guerrero, Carlos Barrera-Avalos, Silvia Matiacevich, Leonel E. Rojo, Jorge Pavez, Edison Salas-Huenuleo, Marcelo J. Kogan, Alejandro Escobar, Luis A. Milla, Ricardo Fernandez, Mónica Imarai, Eugenio Spencer, Juan Pablo Huidobro-Toro, Claudio Acuña-Castillo

**Affiliations:** ^1^Departamento de Biología, Facultad de Química y Biología, Universidad de Santiago de Chile (USACH), Alameda, 3363 Santiago, Chile; ^2^Centro de Biotecnología Acuícola, Universidad de Santiago de Chile (USACH), Alameda, 3363 Santiago, Chile; ^3^Centro de Nanociencias y Nanotecnología, Universidad de Santiago de Chile (USACH), Chile; ^4^Departamento de Ciencia y Tecnología de los Alimentos, Facultad Tecnológica, Universidad de Santiago de Chile (USACH), Alameda, 3363 Santiago, Chile; ^5^Departamento de Química de los Materiales, Facultad de Química y Biología, Universidad de Santiago de Chile (USACH), Alameda, 3363 Santiago, Chile; ^6^Laboratorio de Nanobiotecnología y Nanotoxicología, Departamento de Química Farmacológica y Toxicológica, Facultad de Ciencias Químicas y Farmacéuticas, Universidad de Chile, Chile; ^7^Advanced Center for Chronic Diseases, Universidad de Chile, Chile; ^8^Instituto de Investigación en Ciencias Odontológicas, Facultad de Odontología, Universidad de Chile, Chile; ^9^Centro de Investigación Biomédica y Aplicada (CIBAP), Escuela de Medicina, Facultad de Ciencias Médicas, Universidad de Santiago de Chile, Chile; ^10^Departamento de Salud, Universidad de Los Lagos, Osorno, Chile

## Abstract

Oncolytic virus therapy has been tested against cancer in preclinical models and clinical assays. Current evidence shows that viruses induce cytopathic effects associated with fusogenic protein-mediated syncytium formation and immunogenic cell death of eukaryotic cells. We have previously demonstrated that tumor cell bodies generated from cells expressing the fusogenic protein of the infectious salmon anemia virus (ISAV-F) enhance crosspriming and display prophylactic antitumor activity against melanoma tumors. In this work, we evaluated the effects of the expression of ISAV-F on the B16 melanoma model, both *in vitro* and *in vivo*, using chitosan nanoparticles as transfection vehicle. We confirmed that the transfection of B16 tumor cells with chitosan nanoparticles (NP-ISAV) allows the expression of a fusogenically active ISAV-F protein and decreases cell viability because of syncytium formation *in vitro*. However, the *in vivo* transfection induces a delay in tumor growth, without inducing changes on the lymphoid populations in the tumor and the spleen. Altogether, our observations show that expression of ISAV fusion protein using chitosan nanoparticles induces cell fusion in melanoma cells and slight antitumor response.

## 1. Introduction

Oncolytic virus therapy is an emerging promissory option for cancer treatment. It uses competent replicating viruses as tools to destroy cancer cells. It was initially derived from serendipitous observations that viral infections caused remission in numerous malignancies. The ability to efficiently induce cellular destruction in tumor cells is associated with impaired genome repairing mechanisms, which allow unregulated viral replication, as well as alteration of immune response mechanisms [1]. In normal cells, these protective responses keep viral replication under control, but fusogenic oncolytic viruses waive these protective responses, and this is why they are considered promising candidates to enhance immunotherapy and initiate broad antitumor responses. These viruses display cytopathic effects through syncytium formation mediated by the expression of viral fusion proteins [2]. Syncytium is viable for short time periods, and evidence suggests that it undergoes immunogenic cell death (ICD) [3]. Virus-cell fusion is achieved by one or more viral surface glycoproteins, denoted as fusogenic membrane glycoproteins (FMGs) or simply fusion proteins. FMGs mediate virus entry into the cell by a fusion process between the viral membrane and the cellular membrane. FMGs also trigger fusion between the viral membrane and endosomal membranes if protein activity requires posttraductional modifications in an acid environment. Although a series of clinical trials with oncoviral therapy in humans have been carried out since the 1970s, it was only in 2015 that the herpes-mediated therapy was approved by the FDA for the treatment of unresectable stages of melanoma [4]. Additional oncolytic viruses and their uses in combination with other treatments are currently under investigation [5]; several of them are already at the level of clinical trials with promising effects [3, 6]. Some of these viruses promote efficient malignant cell death, employing direct and/or immune-mediated mechanisms [7, 8].

Viral modifications are necessary to develop safe and effective cancer immunotherapies, which is time-consuming and rather expensive to scale-up [3]. The use of viral fusion proteins has been proposed as an alternative to the use of complete viruses. In theory, transfection of tumor cells expressing viral fusion proteins might trigger syncytium formation and, therefore, destruction of tumor cells and activation of antitumor immune responses [3]. For instance, *in vitro* expression of the fusion gibbon ape leukemia virus (GALV-F) protein in human tumor models of hepatocarcinoma, melanoma, and fibrosarcoma caused cell death. This process was mediated by syncytium formation and the release of exosome-type extracellular vesicles, called syncytiosomes, which can be recognized by immune cells triggering an antitumor response [9–11]. Similarly, intratumor treatment in immunodeficient mice bearing human lung carcinoma-derived tumors, with the gene encoding the GALV-F protein or the F protein of the endogenous human retrovirus W (HERV-W), shows significant delay in tumor growth [12].

Additionally, the use of replication-defective adenovirus containing active fusogenic proteins against syngeneic bilateral colon cancer resulted in improved survival and systemic antitumor immune responses in mice [13]. Similar results were observed when expressing the fusion protein G from vesicular stomatitis virus (VSV-G) in B16 melanoma cells, improving the efficacy of a weak allogeneic vaccine [14]. Also, the expression of the fusion protein of human respiratory syncytial virus (hRSV) in a murine colon cancer model led to potent local antitumor effect and cell fusion [13]. Interferon-sensitive VSV encoding reovirus fusion protein exhibited increased activity against two models of breast cancer in vitro (MCF-7 and 4T-1) and extending survival of 4T1 metastatic mammalian cancer and CT26 metastatic colon cancer animals *in vivo* [15]. The results of this series of studies show that the expression of viral fusion proteins in tumor cells would kill cancer cells through syncytium formation and subsequent release of syncytiosomes loaded with tumor antigens, which in turn would trigger an antitumor response [3, 11, 16].

The use of dying cells or extracellular vesicles from apoptotic cells generated from tumor cells expressing FMGs has also been evaluated as antitumoral therapy. The syncytiosomes released *in vitro* by human melanoma cells (Mel888 and Mel624) expressing the GALV-F protein act as a tumor antigen source, activating CD8+ T lymphocytes by crosspresentation of gp100, a melanoma-associated antigen [11, 16]. Recently, our group reported that immunization with dead tumor cells expressing the fusion protein of the infectious salmon anemia virus (ISAV-F) facilitates an antigenic presentation and generates dendritic cell maturation. However, it fails to induce an effective antitumor response *in vivo*. In the present study, we evaluated a new transfection strategy based on chitosan nanoparticles for *in situ* transfections of murine melanoma tumors with the ISAV fusion protein gene. We propose this strategy based on the biocompatibility, biodegradability, and innocuousness of chitosan as a pharmaceutical excipient for efficient drug delivery [17, 18].

## 2. Materials and Methods

### 2.1. Nanoparticle Generation and Characterization

Complexes were generated by the coacervation method [19] using chitosan (75-85% of deacetylation degree and 50-190 kDa, Sigma) and the vectors described for each case. Chitosan at 0.25% in acetic acid, pH 5.5, was mixed with plasmid DNA (pDNA) dissolved in MilliQ water and incubated 5 min at 55°C and then maintained for 30-60 min at room temperature until its use. pDNA was kept constant, and the amount of chitosan was varied, generating N/P ratios (-NH_2_ group of chitosan versus -PO_4_^2-^ group of pDNA) of 1/4, 1/20, 1/28, and 1/40. To characterize the chitosan/pDNA complexes, we made different evaluations. First, we determined complex formation by the electrophoretic migration delay of the pDNA complexed by the chitosan in comparison to naked pDNA (NV, naked vector), using a 0.8% agarose gel in TAE buffer, stained with Gel Red (Biotium). Second, the pDNA loading efficiency was determined by calculating the percentage of pDNA complexed, based on the amount of initial pDNA used to generate the complexes (2.5 *μ*g) and the amount of pDNA present in the supernatant after centrifugation at 14000 g for 10 min. The pDNA was quantified using the Tecan Infinite 200pro spectrophotometer. Third, we determined the diameter and the zeta potential value, as a measure of the surface charge, of the complexes in a 1 ml suspension, using the Zetasizer Nano ZS equipment (Malvern Panalytical, United Kingdom). Fourth, we evaluated the morphology of the suspended complexes synthesized at an N/P ratio of 1/20 and 1/28 by atomic force microscopy (AFM) with the NanoScope IIIa Multimode Equipment (Digital Instruments, USA). Finally, using an expression vector with the green fluorescence protein (GFP) coding sequence as a reporter, we evaluated the transfection efficiency of the different pDNA/complexes in B16 cells after 48 hours, determining GFP+ B16 cells. Samples were analyzed using the Accuri C6 Flow Cytometer (BD Biosciences, USA), and the information was processed using CFlow Plus software (BD Biosciences).

### 2.2. Transcript Expression

The coding sequence of the ISAV-F protein was subcloned from a pUC57 vector into the commercial expression vector for eukaryotic cells, pIRES2 (BD Biosciences Clontech, PT3267-5) as described previously (Morales et al., 2017).

ISAV-F mRNA expression in B16 cells after 48 hours posttransfection with nanoparticles at N/P ratio of 20 was evaluated by conventional RT-PCR. Briefly, the cells were harvested, and the total RNA was extracted with Trizol® Reagent (Gibco, 15596026) according to the manufacturer's recommendations. Subsequently, 1 *μ*g of total RNA was treated with DNase (RQ1 DNase free of Rnase; Promega, M610A) for 30 min at 37°C and then used for cDNA synthesis using reverse transcriptase M-MLV and OligodT15 (Promega, C1101) according to the manufacturer's instructions. The transcripts of ISAV-F were detected by PCR (Fw 5′-ATCGAAGCTTATGGCATTCCTGACTAT-3′ and Rv 5′-CCTGGTGCACTTCGGACG-3′), detecting the transcript of the enzyme glyceraldehyde 3-phosphate dehydrogenase (GAPDH, Fw 5′-TCGGTGTGAACGGATTTGGC-3′ and Rv 5′-TTTGCCGTGAGTGGAGTCATACTG-3′) as control of constitutive expression. The product obtained was observed on a 1% agarose gel staining with Gel Red (Biotium, 41002).

### 2.3. Cell Fusion

Cell fusion was further confirmed by evaluating the presence of syncytium in transfected B16 cells. Briefly, B16 cells were seeded on coverslips. When 40-60% confluence was achieved, were transfected with 0.5 *μ*g of the pIRES-ISAV plasmid using Lipofectamine 2000 (Invitrogen, 11668027) according to manufacturer recommendations and with 2.5 *μ*g of the same plasmid present in chitosan NPs synthesized at N/P 20. At 48 hours posttransfection, the cells were washed with PBS and incubated with the CellMask Orange plasma membrane stain probe (Life Technologies, C10045) according to the manufacturer's recommendations, fixed with 3.75% paraformaldehyde for 30 min at 37°C and finally incubated with DAPI 0.5 mg/ml for 5 min. Covers were mounted on slides with DABCO and observed using LSM 800 Zeiss confocal microscope. Numbers of syncytia were quantified in B16, B16 Lipo-ISAV, and B16 NP-ISAV and expressed as number by field.

### 2.4. Cytotoxicity

The effect in cell viability produced by the ISAV-F expression was evaluated by measuring metabolic activity in transfected B16 cells. To do so, 3.5 × 10^4^ B16 cells were transfected with chitosan nanoparticles or Lipofectamine 2000 under the same conditions mentioned above. After 24, 48, and 120 hours, MTT (Sigma-Aldrich, M2128) assays were performed in agreement with manufacturer suggestions. The absorbance value at 570 nm of nontransfected cells was used as a 100% viability value.

### 2.5. Animals

Mice of 8-10 weeks of transgenic strain C57BL/6J FoxP3^GFP+^ expressing GFP under the control of FoxP3 promoter in Treg cells were obtained from the animal facilities of Facultad de Química y Biología from Universidad de Santiago de Chile. The animals were maintained with *ad libitum* feeding under 12/12 light and dark cycles. The experimental animal protocols were approved by the Bioethics Committee of the Universidad de Santiago de Chile (Letter No. 489).

B16 cell suspensions were used to induce tumor development in C57BL/6J FoxP3^GFP+^ strain by subcutaneous (s.c.) injection of 2 × 10^5^ living cells in the mouse lumbar region (challenge). Animals were separated into three groups when the tumor size reached 2.0 mm^3^, detectable tumors appeared between days 9 and 17 after challenge. The tumor distribution in all animals and the occurrences of tumors among the experimental groups were not significantly different. The first group did not receive treatment (control), the second group was treated with chitosan (CH), and the third group was treated with NPs of chitosan and pIRES-ISAV (NP-ISAV). Chitosan treatment consisted of an intratumoral (i.t.) injection of 122 *μ*g of chitosan in 100 *μ*l of PBS. The NP-ISAV treatment consisted of an i.t. injection of a nanoparticle suspension synthesized at an N/P 20 composed of 122 *μ*g of chitosan and 10 *μ*g pIRES-ISAV in 100 *μ*l of PBS. Tumor growth was evaluated by measuring the tumor size using a caliper and calculating tumor volume according to the half-sphere formula (*V* = 2/3*πr*^2^, where *V* corresponds to the volume in mm^3^ and *r* is the tumor radius in mm). A maximum tumor volume (MTV) of 260 mm^3^ was used as the endpoint criterion, where animals were sacrificed by cervical dislocation and processed for subsequent analyses.

### 2.6. Splenocyte and Infiltrating Tumor Lymphocyte Evaluation

After euthanasia, the spleen and tumor of C57BL/6 FoxP3^GFP+^ mice were extracted. The spleen was disaggregated in a 100-mesh metal grid and then treated with ACK buffer (155 mM NH_4_Cl, 10 mM KHCO_3_, and 1 mM Na_2_EDTA, pH 7.3) for 5 min with gentle shaking to remove, by differential lysis, erythrocytes. In parallel, the tumor was cut and treated with Trypsin (HyClone, SH30042.01) in RPMI 1640 medium (Gibco, 31800022), for 30 min at 37°C with constant agitation. After this, to collect the cells, the solid tumor remains were eliminated and the remaining solution, along with the obtained from spleen, was centrifuged at 1600 g for 5 min. Splenocytes and tumor cells were resuspended in RPMI 1640 medium (Gibco, 31800022) with 10% fetal bovine serum (BFS) (Biological, DW105804-127-1A). For splenocyte labeling, 2 × 10^6^ cells were used, and for tumor cells, 150 *μ*l of the cell suspension previously obtained. All antibody labeling was performed for 30 min at 4°C. For CD8+ and CD4+ population labeling, Anti-Mouse CD8a-PE (eBioscience, Clone: 53-6.7) and Anti-Mouse CD4-PE (eBioscience, Clone: GK1.5) were used, respectively. For the CD4+ subpopulations, the cells were fixed and permeabilized with Fix-Perm buffer (intracellular fixation permeabilization buffer set, eBioscience, 88-8824-00) for 30 min at 4°C and subsequently labeled; for CD4+ ROR*γ*t (Th17), the Anti-Mouse ROR*γ*t-APC (eBioscience, Clone B2D); and for CD4+ Tbet (Th1), the Anti-Human/Mouse Tbet-PE-Cyanine7 (eBioscience, Clone: eBio4B 10). Finally, the FoxP_3_ CD4+ subpopulation (Treg) was determined using the FoxP3-GFP labeling of the reporter mice used. For the data acquisition, the BD Accuri C6 equipment (BD Bioscience, USA) was used, and for the data analysis, the FlowJo 7.6.1 software was used.

### 2.7. Statistical Analysis

Results were plotted and presented as average value ± SEM and analyzed by the nonparametric Mann–Whitney test. A confidence value of 95% was used. All analyses were performed using GraphPad Prism 5.01 software (GraphPad Software, Inc., USA).

## 3. Results

### 3.1. Nanoparticle Characterization

**Figure 1:**
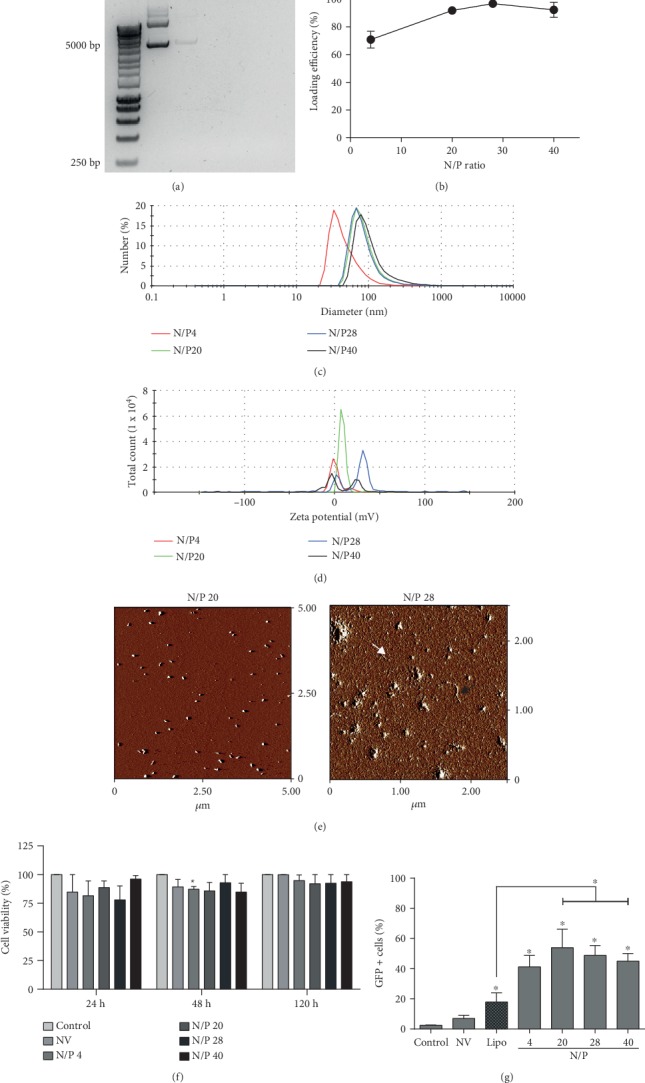
NP characterization and evaluation. (a) Complex ensemble was evaluated at various ratios between chitosan and pGFP by electrophoretic migration of pDNA in the presence of chitosan on an agarose gel. DNA ladder (L) is shown in lane 1, naked vector (NV) was used as a negative control in lane 2, and the different N/P complexes are shown in lanes 3-6. (b) Complex formation efficiency was evaluated as described in Materials and Methods, and it corresponds to insoluble pDNA with respect to total pDNA and represented as average+/−standard error. Representative histograms from physical parameters such as (c) size and (d) zeta potential for N/P 4, 20, 28, and 40 are shown in red, green, blue, and black, respectively. (e) NP morphology was determined by atomic force microscopy for N/P 20 and 28. (f) The effect of the naked vector (NV) and NP (N/P from 4 to 40) on cell viability was evaluated by MTT assays at 24, 48, and 120 h after challenge and was normalized against untreated cells (control). (g) Transfection efficiency was evaluated using GFP as a reporter with NPs or Lipofectamine and evaluated by flow cytometry. A minimum of three independent experiments was performed. Bars correspond to average+/−standard error, and statistical analyses were performed using the Mann–Whitney test (^∗^*p* < 0.05).

The formation of complexes between chitosan and pGFP was evaluated by electrophoretic migration of pDNA in the presence of chitosan in an agarose gel. In the case of the complexes synthesized at N/P ratio of 1/4, the pDNA band pattern obtained was similar to the control without chitosan (Figure 1(a)), suggesting that part of the pGFP was free and did not interact with the chitosan. In contrast, in the complexes synthesized in the presence of higher levels of chitosan (N/P ratios of 1/20, 1/28, and 1/40), no migration of pGFP was observed; this is the evidence of their complete interaction with the polysaccharide (Figure 1(a)). In order to measure the efficiency in complex formation, we quantified the loading efficiency, which accounts for the amount of pGFP complexed by chitosan. We observed differences in loading efficiencies according to the relative amount of chitosan. Complexes synthesized with the lowest relative amount of chitosan (N/P ratio of 1/4) were able to complex 70% of the available pDNA. However, higher loading efficiencies of 92%, 97%, and 93% were achieved when N/P ratios were increased to 1/20, 1/28, and 1/40, respectively (Figure 1(b)).

Once the formation of the complexes was confirmed, physical parameters such as size, surface charge (determined by zeta potential), and morphology were analyzed. We observed a monodisperse diameter in complexes synthesized at N/P ratio of 1/4, evidenced by the presence of a single peak at 32.7 nm in the distribution graph. Similar behavior was observed in complexes at N/P ratio of 1/20 and 1/28, with a peak at 68.1 nm in diameter and complexes at N/P ratio of 1/40 with a peak at 78.8 nm (Figure 1(c)). This evidence supports the fact that the diameter of all chitosan/pGFP complexes was below 100 nm, which meets the criteria for the nanoparticle (NP) classification.

The surface charge of the NPs was evaluated by measuring zeta potential. At N/P ratio of 1/4, NPs showed monodispersion with a peak at -1.22 mV, while at N/P ratios of 1/28 and 1/40, two peaks were observed (N/P ratio of 1/28: 2.08 and 31.8 mV; N/P ratio of 1/40: -2.81 and 23.2 mV), indicating a heterogeneous NP population. In contrast, the nanoparticles of N/P ratio 1/20 also showed a homogeneous distribution with a single positive peak close to 7.15 mV (Figure 1(d)). The positive zeta potential value suggests that NPs have a positive surface charge. This feature increases its solubility in aqueous medium and facilitates its interaction with the cellular membranes.

The final step in the NP physical characterization was the determination of their morphology by atomic force microscopy. NPs synthesized at N/P ratio of 1/20 displayed homogeneous spherical type morphology. However, increasing the amount of chitosan up to an N/P ratio of 1/28 yielded spherical nanoparticles and free chitosan fibers (Figure 1(e)), evidencing an excess of chitosan.

To determinate a possible cytotoxic effect of the nanoparticle in culture cells, cell metabolic activity was evaluated at 24, 48, and 120 hours posttreatment on B16 tumor cells. None of the evaluated conditions showed significant differences in the viability of the cells treated with nanoparticles compared with the untreated control group (Figure 1(f)). These results demonstrate that the tested dosage range of chitosan-pGFP nanoparticles is not toxic for cultured B16 cells.

Finally, the functionality of the NPs was evaluated measuring their transfection capacity in the B16 cells, quantifying the percentage of GFP-positive cells (GFP^+^) by flow cytometry. All the NPs evaluated showed over 40% of GFP^+^ cells, being in all cases significantly higher than control nontransfected cells. Interestingly, N/P ratio of 1/4 showed significantly even higher efficiency than those cells transfected with Lipofectamine (18%) (Figure 1(g)).

### 3.2. Expression of the ISAV-F Protein in B16 Cells

**Figure 2:**
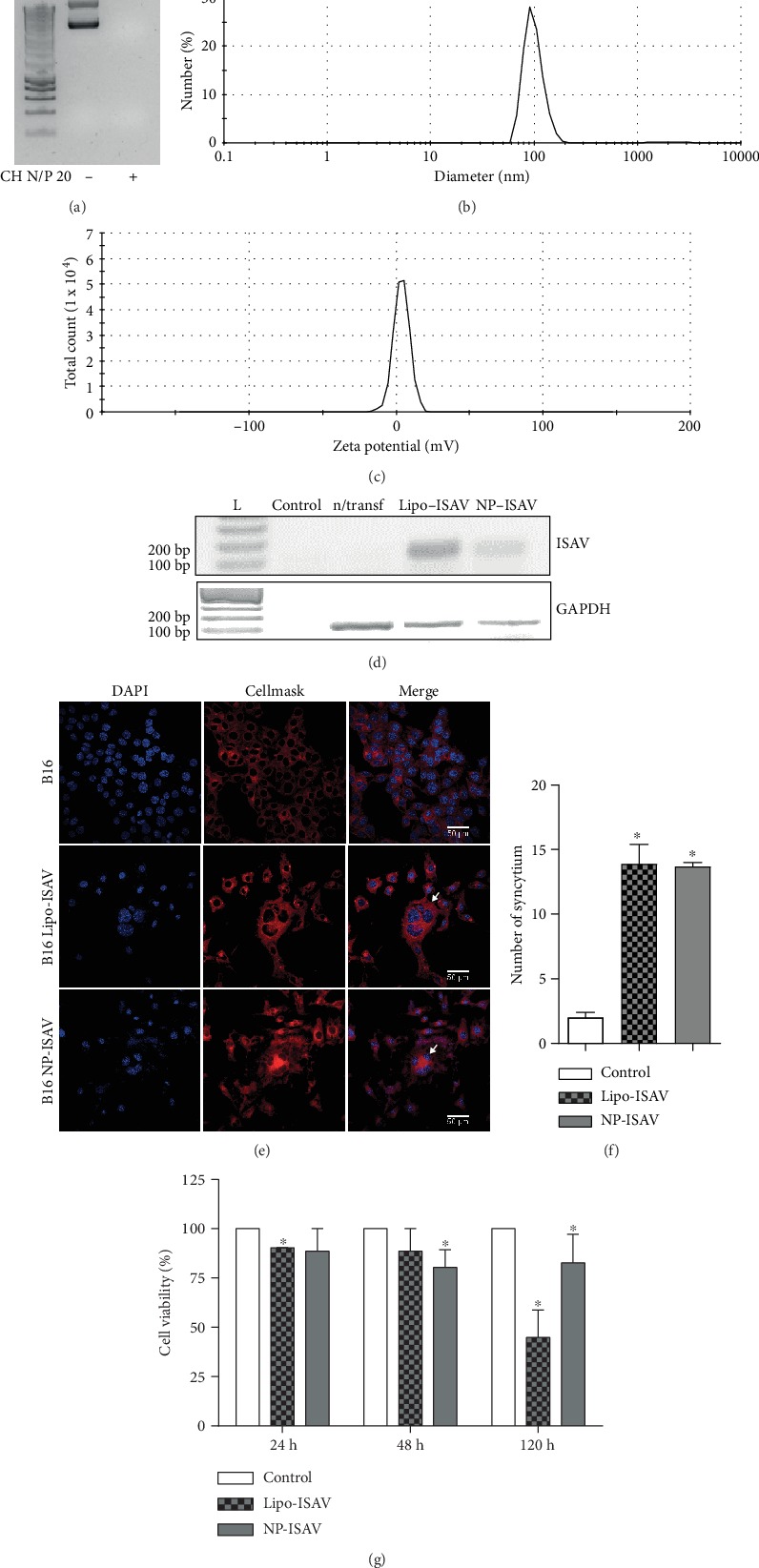
NP-ISAV characterization and effects. (a) Complex ensemble was evaluated between chitosan and pIRES-ISAV vector by electrophoretic migration on an agarose gel. DNA ladder (L) is shown in lane 1, naked vector in lane 2, and complexes in lane 3. Representative histograms from physical parameters such as (b) size and (c) zeta potential are shown. (d) The expression of ISAV fusion protein (upper panel) and housekeeping GAPDH (lower panel) was determined by RT-PCR 48 hours posttransfection. A representative gel shows DNA ladder (L, lane 1), PCR blank control (Blank, lane 2), parental cells nontransfected (n/transf, lane 3), Lipofectamine-ISAV cells transfected (Lipo-ISAV, lane 4), and NP-ISAV cells transfected (NP-ISAV, lane 4). (e) The fusogenic activity of the ISAV-F protein was determined by evaluating the presence of syncytia 48 h posttransfection comparing B16 parental cells (upper panel), Lipo-ISAV transfected B16 cells (middle panel), and NP-ISAV transfected B16 cells (lower panel). Cells were stained with DAPI (left column) and CellMask (middle column). The merge of both colors is shown in the right column, where a white arrow indicates a syncytium. (f) Numbers of syncytia should be quantified in B16, B16 Lipo-ISAV, and B16 NP-ISAV and correspond to the average of 5 fields, of 3 independent experiments. (g) The effect of transfection on cell viability was evaluated at 24, 48, and 120 h posttransfection and was normalized against nontransfected cells; data from Lipofectamine (Lipo-ISAV) and nanoparticle (NP-ISAV) transfected cells were graphed as average+/−standard error. Statistical analyses were performed using the Mann–Whitney test (^∗^*p* < 0.05).

Our main goal was to confirm the fusogenic activity that was previously described for ISAV in mammalian cells (Morales et al., 2017) in this case, with chitosan nanoparticles as low toxicity and high-efficiency transfection method [20]. Based on our NP characterization results, chitosan/pIRES-ISAV complexes were synthesized at N/P ratio of 1/20, as described in Materials and Methods. Under these conditions, we observed a delayed electrophoretic migration of the pIRES-ISAV vector, indicating a successful complexation interaction with chitosan (Figure 2(a)). These complexes showed an average nanoparticle diameter close to 100 nm (Figure 2(b)) and a positive superficial charge with a zeta potential value of 5.57 mV (Figure 2(c)). The expected amplicon corresponding to the ISAV transcripts was detected 48 hours posttransfection using NP-ISAV (chitosan/pIRES-ISAV nanoparticles) in B16 cells. The same was observed when we used Lipofectamine (Lipo-ISAV), but not in nontransfected cells (Figure 2(d)). Altogether, these results indicate that the use of NP-ISAV as transfection method allows the expression of the ISAV-F transcript in cultured B16 cells.

The fusogenic activity of the ISAV-F protein was determined by evaluating the presence of syncytia 48 h posttransfection of B16 cells. At this time, we observed large multinucleated cells, a clear sign of successful syncytium formation in NP-ISAV transfected cells. This phenomenon was also observed in Lipo-ISAV transfected cells, but not in nontransfected cells (Figure 2(e)). Syncytium numbers were quantified in B16, B16 Lipo-ISAV, and B16 NP-ISAV as described in Materials and Methods. Regardless of the transfection methodology, ISAV induced a significantly higher number of syncytia in all tested conditions (Figure 2(f)). In NP-ISAV transfected cells, the syncytia observed were associated with a 20% decrease in B16 cell viability between 48 and 120 hours posttransfection (Figure 2(g)). Altogether, these results show that the transfection of B16 tumor cells with NP-ISAV allows the expression of a fusogenically active ISAV-F protein and decreases cell viability associated with syncytium formation.

### 3.3. Effect of Melanoma NP-ISAV Intratumor Treatment

**Figure 3:**
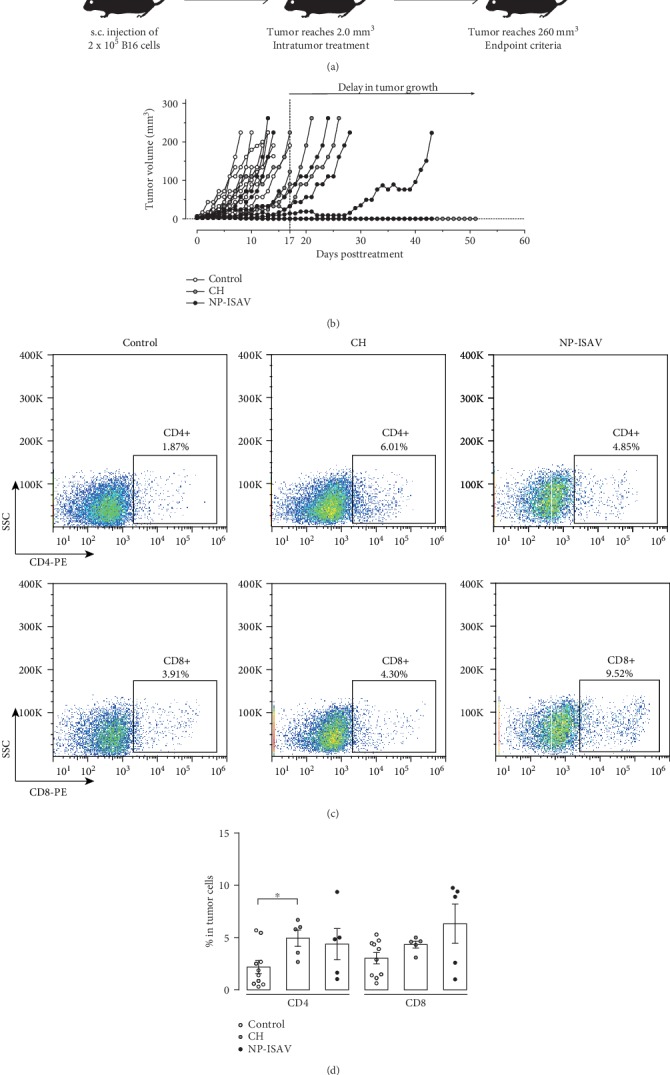
Effect of treatment with NP-ISAV on tumor growth. (a) Treatment diagram. Mice were injected subcutaneously (s.c.) with 2 × 10^5^ living cells in the mouse lumbar region. When the tumor reached 2.0 mm^3^, mice received, or not (control group), chitosan or NP-ISAV intratumor treatment and were daily monitored until the endpoint criteria. (b) Tumor growth was compared between the nontreated group (control, open circles), the chitosan-treated group (CH, gray circles), and the NP-ISAV-treated group (NP-ISAV, black circles). Representative histograms from tumor-infiltrating T (c) CD8+ (lower panel) and CD4+ (upper panel) cells are shown for untreated mice (control), chitosan treated (CH), or chitosan ISAV nanoparticles (NP-ISAV). (d) A summary of at least 5 independent experiments is shown. Bars correspond to average+/−standard error. Individual experiments are graphed; statistical analyses were performed using the Mann–Whitney test (^∗^*p* < 0.05).

We previously reported that a specific type of cell body generated from B16 cells expressing ISAV-F induces antigen presentation *in vitro*, but does not generate antitumor responses in immunized mice with melanoma [21]. Based on this finding, we aim to determine the *in vivo* effect on tumor growth of murine B16 melanoma treated with an intratumor (i.t.) injection of NP-ISAV. Mice challenged with B16 cells showed a detectable tumor between days 9 and 17 postchallenge (Figure 3(a)). NPs were injected i.t. when the tumor reached 2 mm^3^ volume, and the tumor was monitored daily until it reached a maximal volume (MTV) of 260 mm^3^. The tumor growth of treated animals was compared with their control counterparts (without treatment). We observed that NP-ISAV treatment induced a delay in tumor growth in three mice and a complete regression of the tumor in one mouse (Figure 3(b)). CH treatment induced a delay in two mice and complete regression in one mouse. However, no significant differences were observed in the group treated with chitosan, with respect to the NP-ISAV-treated group.

**Figure 4:**
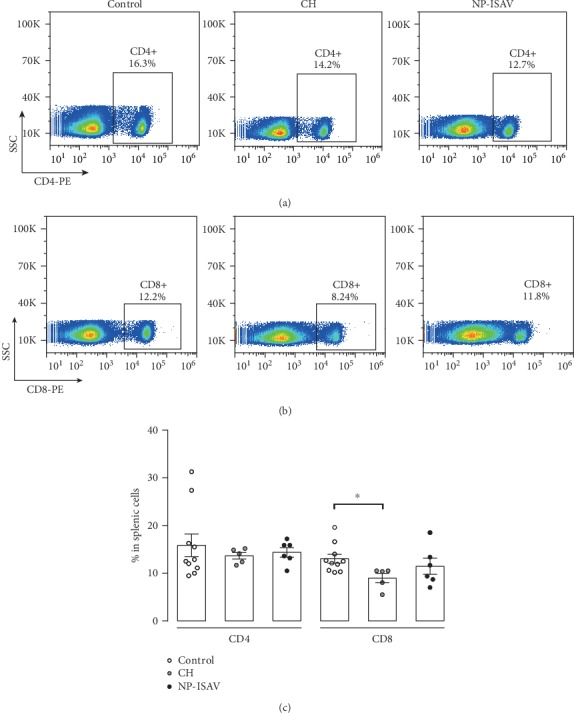
Effect of treatment with NP-ISAV on splenic T cells. Representative histograms of splenic T (a) CD8+ and T (b) CD4+ cells are shown for untreated mice (control), chitosan treated (CH), and chitosan ISAV nanoparticles (NP-ISAV). (c) A summary of at least 5 independent experiments is shown. Bars correspond to average+/−standard error; individual experiments are graphed; statistical analyses were performed using the Mann–Whitney test (^∗^*p* < 0.05).

**Figure 5:**
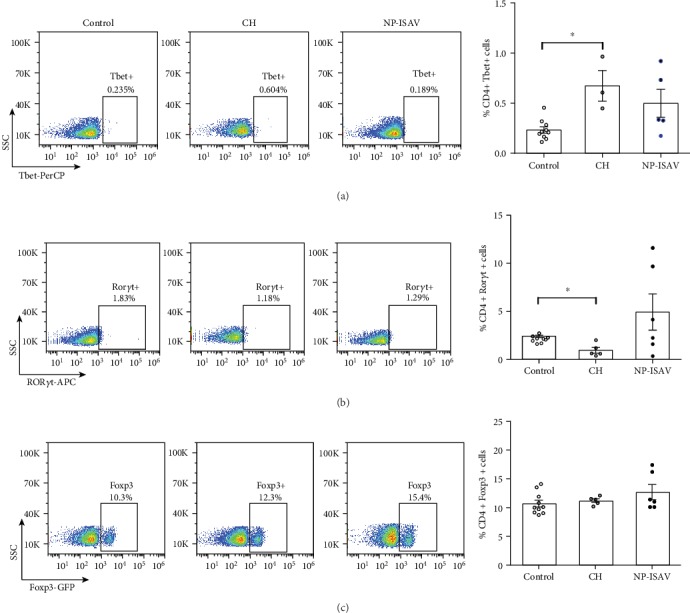
Effect of treatment with NP-ISAV on splenic T CD4+ subpopulations. Splenic (a) CD4+ Tbet+, (b) CD4+ ROR*γ*t+, and (c) CD4+ FoxP_3_+ subpopulations were determined as described in Materials and Methods for untreated mice (control), chitosan treated (CH), and chitosan ISAV nanoparticles (NP-ISAV). Left corresponds to representative histograms and right corresponds to a summary of at least 5 independent experiments. Bars correspond to average+/−standard error; individual experiments are graphed; statistical analyses were performed using the Mann–Whitney test (^∗^*p* < 0.05).

A possible explanation for our observations on the tumor growth delay could be associated with the induction of an immune response against the tumor, according to results observed in immunized mice with dead B16 cells expressing ISAV-F [21]. We did not observe significant changes in CD4+ and CD8+ infiltrating lymphocyte populations in the tumors (Figure 3(c)) and in the spleen (Figure 4). Similarly, no changes were detected in CD4+ in spleen expressing Tbet, ROR*γ*t, and Foxp3, in response to the treatment with NP-ISAV, and only a significant increase of splenic Tbet+ cells after chitosan treatment was observed (Figure 5). Altogether, our results suggest that intratumor NP-ISAV treatment induces significant tumor delay, probably associated with cell fusion induction.

## 4. Discussion

We determined that intratumoral treatment of murine B16 melanoma with ISAV fusion protein gene-containing chitosan nanoparticles delayed tumor growth and induced complete regression of the tumor in one case. This work is to our knowledge the second report of the fusogenic activity of the ISAV-F protein in mammalian cells. The first work was published by our group [21] and led us to propose an improvement in the antitumor efficacy through a chitosan nanoparticle-based intracellular delivery of viral fusion protein genes.

According to evidence, the antitumor effect associated with viral fusion proteins is based on the destructive function of these proteins and the action of the immune system. Although ISAV-F-mediated effects could be explained in several ways, we believe that direct cytopathic effects are key for its intratumoral effect, because of the expression of GALV and HERV-W fusion proteins in human lung carcinoma tumors [12]. In this cancer model system, a highly fusogenic variant of the GALV-F protein induces the formation of unstable syncytia of short half-life, which increases cytotoxicity [10, 12] and inhibits tumor growth *in vivo* [12, 16]. Most studies of the antitumor effect of viral fusion proteins have been based mainly on the action of a highly fusogenic variant of the GLAV-F protein, which is part of the envelope of the gibbon ape leukemia virus and is responsible for mediating fusion between the viral membrane and the target cell membrane. The expression of GALV-F in H322 and A549 cells of human lung carcinoma produces 55.5% and 78.9% decrease in cell viability at days 4 and 5 posttransfection, respectively [12]. This cytotoxic effect is attributed to the formation of multinucleated or syncytial cells derived from cell fusion mediated by GALV-F. These syncytia are initially viable and remain active both metabolically and transcriptionally [10]. However, they eventually lose their adherence and die, by a type of cell death that has not yet been fully characterized. Actually, apoptotic [22] and nonapoptotic cell death [11] have been documented in recent reports. Even though we did not observe an aggressive formation of syncytium *in vitro* after ISAV-F expression, a cytotoxic effect was definitely present. Therefore, we cannot rule out this possible mode of action *in vivo*.

Furthermore, syncytia-associated cell death corresponds to a highly immunogenic type of cell death, characterized by the release of several extracellular vesicles named syncytoms, which contain tumor antigens [11]. These syncytoms have proved to be an efficient antigenic source for the maturation and activation of antigen-presenting cells. These cells activate CTL lymphocytes, which could lead to the assembly of antitumor immune responses *in vivo* [14, 23–25]. Consequently, we hypothesized that dead B16 melanoma cells expressing the ISAV-F, induced by deprivation of nutrients, are an efficient antigenic source that induces DC maturation and antigen crosspresentation *in vitro*. Furthermore, preventive immunization with CBs-B16-ISAV in mice induces a delay in tumor growth of B16 melanoma associated with an increase in splenic CD4+ and CD8+ T lymphocyte populations [21]. Although, in this work, NP-ISAV treatment did not induce significant changes in the analyzed lymphocyte populations, the time point at which we measured these changes could explain these results, as possible changes on T cell populations induced by the treatment could have been already overcome at the time we measured it (when MTV was achieved) [26, 27]. It is indeed possible that the absence of changes reflects an antitumor immune response at late phases, whose action would have been overcome by the tumor cells leading to tumor growth. Also, different cell types may play a role in this type of response, such as natural killer (NK) lymphocytes [28].

Chitosan has been evaluated for its antitumor activity since the 1980s. Oral administration of oligoderivatives of chitosan in mice with solid tumors of Sarcoma 180 [29] and by intravenous administration in mice with Meth-A tumor [30] induced tumor growth inhibition, increase levels of IL-1 and IL-2, and also a polarization to cytotoxic T lymphocytes, in the case of Meth-A. Since then, numerous reports have been published on the use of chitosan as a vehicle for small molecules against different forms of cancer by oral, intragastric, intravenous, and intraperitoneal administration [31, 32]. Also, intraperitoneal treatment with chitosan triggers an antitumor immune response in the murine B16 melanoma model through the action of dendritic cells leading to the activation and action of IFN*γ* secreting NK lymphocytes [28]. These results point to a possible link between chitosan and the immune system activation towards the production of IFN*γ*. *In vitro* assays have shown that chitosan activates dendritic cells [28, 33] and macrophages [34, 35], triggering the release of IL-1*β* and IL-12, crucial cytokines for CD4+ lymphocyte polarization to effector Th1 lymphocytes and to increased levels of IFN*γ* (a potent antitumor cytokine) [36]. In this work, tumor growth delay and complete tumor regression in mice after treatment with chitosan would support the idea that this natural polymer induces an antitumor cell response, probably commanded by Th1 (Tbet+) lymphocytes.

## 5. Conclusion

In summary, chitosan nanoparticles allow the expression of a fusogenically active ISAV fusion protein, which in turn induces cell fusion and cytotoxicity in B16 melanoma cells *in vitro*. However, its use to treat melanoma tumors induced slight *in vivo* antitumoral effect in comparison to chitosan treatment. We believe that further work is necessary, but ISAV-F could be a good alternative/complementary agent for the treatment of tumors with a slow growth rate.

## Data Availability

All the data used to support the findings of this study are available from the corresponding author upon request.
